# Cortical resting motor threshold difference in asleep-awake craniotomy for motor eloquent gliomas: WHO grading influences motor pathway excitability

**DOI:** 10.1093/cercor/bhad493

**Published:** 2023-12-18

**Authors:** Ana M Pescador, José P Lavrador, Alba D Baamonde, Christos Soumpasis, Prajwal Ghimire, José D S Mosquera, Carlos Fiandeiro, Holly Jones, Smita Gosavi, Arjel Lejarde, Emily Lawson, Sian Murace, Richard Gullan, Keyoumars Ashkan, Ranjeev Bhangoo, Francesco Vergani

**Affiliations:** Department of Neurosurgery, King’s College Hospital NHS Foundation Trust, Denmark Hill, London SE5 9RS, United Kingdom; Department of Clinical Neurophysiology, King’s College Hospital NHS Foundation Trust, Denmark Hill, London SE5 9RS, United Kingdom; Department of Neurosurgery, King’s College Hospital NHS Foundation Trust, Denmark Hill, London SE5 9RS, United Kingdom; Department of Neurosurgery, King’s College Hospital NHS Foundation Trust, Denmark Hill, London SE5 9RS, United Kingdom; Department of Clinical Neurophysiology, King’s College Hospital NHS Foundation Trust, Denmark Hill, London SE5 9RS, United Kingdom; Department of Neurosurgery, King’s College Hospital NHS Foundation Trust, Denmark Hill, London SE5 9RS, United Kingdom; Department of Neurosurgery, King’s College Hospital NHS Foundation Trust, Denmark Hill, London SE5 9RS, United Kingdom; School of Biomedical Engineering and Imaging Sciences, King’s College London, London, United Kingdom; Department of Neurosurgery, King’s College Hospital NHS Foundation Trust, Denmark Hill, London SE5 9RS, United Kingdom; Department of Clinical Neurophysiology, King’s College Hospital NHS Foundation Trust, Denmark Hill, London SE5 9RS, United Kingdom; Department of Anesthesia, King’s College Hospital NHS Foundation Trust, London, United Kingdom; Department of Anesthesia, King’s College Hospital NHS Foundation Trust, London, United Kingdom; Department of Anesthesia, King’s College Hospital NHS Foundation Trust, London, United Kingdom; Department of Clinical Neurophysiology, King’s College Hospital NHS Foundation Trust, Denmark Hill, London SE5 9RS, United Kingdom; Department of Clinical Neurophysiology, King’s College Hospital NHS Foundation Trust, Denmark Hill, London SE5 9RS, United Kingdom; Department of Clinical Neurophysiology, King’s College Hospital NHS Foundation Trust, Denmark Hill, London SE5 9RS, United Kingdom; Department of Neurosurgery, King’s College Hospital NHS Foundation Trust, Denmark Hill, London SE5 9RS, United Kingdom; Department of Neurosurgery, King’s College Hospital NHS Foundation Trust, Denmark Hill, London SE5 9RS, United Kingdom; Department of Neurosurgery, King’s College Hospital NHS Foundation Trust, Denmark Hill, London SE5 9RS, United Kingdom; Department of Neurosurgery, King’s College Hospital NHS Foundation Trust, Denmark Hill, London SE5 9RS, United Kingdom

**Keywords:** cortical motor excitability, resting motor threshold, glioma, craniotomy

## Abstract

Developing neurophysiological tools to predict WHO tumor grade can empower the treating teams for a better surgical decision-making process. A total of 38 patients with supratentorial diffuse gliomas underwent an asleep-awake-sedated craniotomies for tumor removal with intraoperative neuromonitoring. The resting motor threshold was calculated for different train stimulation paradigms during awake and asleep phases. Receiver operating characteristic analysis and Bayesian regression models were performed to analyze the prediction of tumor grading based on the resting motor threshold differences. Significant positive spearman correlations were observed between resting motor threshold excitability difference and WHO tumor grade for train stimulation paradigms of 5 (R = 0.54, *P* = 0.00063), 4 (R = 0.49, *P* = 0.002), 3 (R = 0.51, *P* = 0.001), and 2 pulses (R = 0.54, *P* = 0.0007). Kruskal–Wallis analysis of the median revealed a positive significant difference between the median of excitability difference and WHO tumor grade in all paradigms. Receiver operating characteristic analysis showed 3 mA difference as the best predictor of high-grade glioma across different patterns of motor pathway stimulation. Bayesian regression found that an excitability difference above 3 mA would indicate a 75.8% probability of a glioma being high grade. Our results suggest that cortical motor excitability difference between the asleep and awake phases in glioma surgery could correlate with tumor grade.

## Introduction

An estimation of tumor grading at the time of surgery for diffuse gliomas is an important step to tailor a personalized tumor resection. The differences in prognosis of different tumor grades in glioma surgery change the risk–benefit ratio of tumor resection adjacent to eloquent areas.

Gliomas are known to alter the microstructure and excitability of motor pathways (Mirchandani et al. 2020). Transcranial magnetic stimulation (TMS) for pre-surgical mapping in patients with gliomas has shown an altered interhemispheric excitability that correlates well with WHO grade (Lavrador et al. 2020). Moreover, glioblastomas show marked alterations of the cortical excitability and higher interhemispheric resting motor threshold (RMT) ratios than grades 2 and 3 gliomas (Neville et al. 2021). Therefore, studying the functional impacts of gliomas on surrounding neurological tissue may hint toward aggressiveness of infiltration of the tumor and, indirectly, on tumor grade.

Intraoperatively, neuromonitoring and mapping of the motor cortex may provide an insight of these changes using a measurable output of motor function—motor evoked potentials (MEPs). MEPs have the characteristic of being altered by voluntary muscle tone. In this way, during wakefulness, when the underlying muscle tone is higher than under general anesthesia or natural sleep, MEPs may show larger amplitude, shorter onset latency, prolonged duration, and lower RMT (Valls-Solé et al. 1994; Brum et al. 2016).

The asleep-awake-sedated surgical protocol for tumor resection offers a unique opportunity to study the properties of the motor cortex in patients undergoing glioma surgery. Here, we measure the excitability difference in cortical motor thresholds at the level of M1 in patients with diffuse gliomas in asleep and awake phase of surgery and establish a correlation with the WHO grading of diffuse gliomas. We hypothesize that a higher WHO grading would be responsible for an increased alteration of neurophysiological properties of the motor pathway and, therefore, a greater difference in the motor excitability between the asleep and awake phases.

## Materials and methods

Single center retrospective cohort study of patients admitted for surgical resection of supratentorial diffuse gliomas under an asleep-awake-sedated protocol at our institution between April 2021 and March 2023. Patients with prior motor deficits or supratentorial lesions other than diffuse gliomas were excluded. All patients had tumor in an eloquent motor area, defined as <10 mm from either M1 or CST (Bello et al. 2006; Raabe et al. 2014; Rosenstock 2017).

All patients underwent preoperative navigated transcranial magnetic stimulation (nTMS) (Nexstim, Helsinki, Finland) for motor and language mapping and tractography for dissection of the corticospinal tract (CST, Stealth MEDTRONIC© Minneapolis, Minnesota, United States of America). A start region of interest (ROI) was placed subcortically in the pre-central gyrus and subcortical area under the central sulcus and a middle ROI was placed in the brainstem at the level of the superior cerebellar peduncle (Catani 2008).

Asleep-Awake-Sedation technique using total intravenous anesthesia (TIVA) was used in each procedure. Intraoperative mapping was performed after dural opening to identify the primary motor cortex. When not exposed, image navigation plus nTMS landmarks were used to slide a strip electrode toward the motor hand knob. Correct placement of the strip electrode was confirmed by direct cortical stimulation and phase reversal technique. If no response was seen at 25 mA, this area was considered negative for motor mapping. Motor thresholds to calculate the cortical excitability difference between the asleep and the awake phases of surgery were performed first during the asleep phase and were calculated for trains (anodal, monopolar 250 Hz, 500 ms pulse duration, 0.5 Hz inter-train interval) of 5-4-3 and 2 pulses. We attempted to obtain MEPs with a single pulse but given that we were rarely successful to achieve this under general anesthesia, we finally decided not to study this stimulation protocol. The same protocol was then repeated during the awake phase. This was done at a time where we had a condition of alpha predominant frequencies on the electroencephalography (C4-Fz, C3-Fz) and an increase in muscle tone baseline on the free running electromyography (EMG), without obvious voluntary muscle movements of the patient to minimize spinal facilitation (Brum 2016). Cortical Excitability Difference was calculated as the mathematical difference in thresholds between the asleep and the awake thresholds, for each train (5-4-3-2).

Neuropathology diagnosis was performed in all patients according to the most recent edition of the WHO classification (Louis et al. 2021).

The study protocols were approved by the Department of Neurosurgery, Kings College Hospital as a retrospective analysis of intraoperatively recorded data in quality of a service evaluation. All participants consented for the neurophysiological mapping and monitoring during the surgical procedure as a part of routine informed consent for craniotomy approved by the Department of Neurosurgery, Kings College Hospital. Given the low sample size, the findings gathered in this study should not be considered generalizable.

### Statistical analysis

Statistical analysis was performed using Python 3.0 software, based on google Collab Jupyter Notebook platforms and using the following libraries: Sklearn, Pandas, Numpy, Scipy and Matplotlib. To analyze our data, and given that the data were not normally distributed, we used non-parametric statistical tests (Spearman correlation analysis and Kruskal–Wallis analysis of the median and interquartile ranges (IRs)). Kruskal–Wallis analysis of the medians was performed to assess the difference in excitability difference amongst the groups regarding tumor grade, location of the tumor, presence of oedema, presence of seizures at debut, and sex of the patients. Spearman correlations were performed following this to assess the numerical trend of correlation between RMT excitability difference and tumor grade as well as age and RMT excitability difference.

A receiver operating characteristic (ROC) analysis was performed using STATA 3.1 Software©. We calculated the sensitivity, specificity, positive predictive value (PPV), negative predictive value, and area under the curve (AUC) for each paradigm of stimulation and each difference in threshold (from 0 to above 5 mA). Then, based on the best AUC—AUC > 0.70 were considered for analysis (Hosmer Jr et al. 2013)—for each paradigm of stimulation, we elaborated the best score to predict high grade versus low grade gliomas.

To assess the exact probability of tumor grading based on cortical excitability difference, we developed a Bayesian Network on top of the regular techniques for inferential statistics. The network consisted of a series of chance nodes: antiepileptic drugs, age, sex, location of the tumor (frontal, parietal, insular, temporal), grade of the tumor, and excitability difference (based on the ROC analysis). These nodes were joined by edges determined by probable causal relationship between the nodes based on expert domain knowledge.

This case series has been reported in line with the PROCESS Guidelines.

## Results

A total of 38 patients were included in the study (age range 11–70 yr old, 17 male patients and 21 female): 15 patients with WHO grade 2, 14 patients WHO grade 3, and 9 patients with WHO grade 4. Table 1 shows a summary of the clinical status of the patients preoperatively.

**Table 1:** Demographic characteristics of the study cohort.

	Study cohort
N	38
Sex • Female • Male	21 17
Tumor Location • Frontal • Parietal • Insular • Temporal	20 6 6 6
Previous History of Seizures^a^ • Focal • Generalized	24 7 19
Antiepileptic Medication:^b^ • Levetiracetam • Lamotrigine • Phenitoin • Clobazam • Valproate	19 3 1 1 1
Preoperative Motor Deficit	2
WHO grade: • 2 • 3 • 4	15 14 9

^a^One patient had both focal and generalized seizures.

^b^Three patients were on dual antiepileptic medication and two patients with seizures had no antiepileptic medication.

All patients presented MEPs in the asleep and the awake state to train of 5, 4, and 3 pulse stimulation. During the asleep phase, however, two patients did not present MEPs to a train of 2 pulses. Table 2 shows the absolute RMT medians (asleep and awake phases) in response to the different train stimulation paradigms for grade 2, 3, and 4 patients. No significant differences in the RMTs according to the grades were found. (*P* > 0.05).

**Table 2:** Comparison of the RMTs for different trains of stimulation between asleep and awake states in motor eloquent gliomas with different WHO grades.

	TRAIN OF 5		TRAIN OF 4		TRAIN OF 3		TRAIN OF 2		TRAIN OF 1	
	Asleep	Awake	Asleep	Awake	Asleep	Awake	Asleep	Awake	Asleep	Awake
**GRADE 2**	7.5 IR 3.8	5.6 IR 2.4	8.4 IR 4.5	5.8 IR 2.8	8.5 IR 4.1	6 IR 3.1	9.5 IR 3.9	6.3 IR 2.9	12 IR 13	9.8 IR 7.9
**GRADE 3**	9 IR 1.1	4.7 IR 4.4	9.7 IR 1.1	5 IR 4.7	10.5 IR 2.3	5.5 IR 4.8	12.1 IR 4	7 IR 5	15 IR 7.7	7.8 IR 8
**GRADE 4**	9 IR 4	3.2 IR 2.9	10.5 IR 1	3.3 IR 3.1	10 IR 3.4	5.3 IR 2.9	11.5 IR 3.3	3.1 IR 0.4	15 IR 4.5	6 IR 3

This table shows the median and IR RMTs per group, per train paradigm for both conditions tested (asleep and awake). There are no significant differences were between the different WHO grades (*P* > 0.05) when the absolute value for RMTs in both asleep and awake states are considered. When we consider the absolute differences between both states (asleep versus awake) the difference is significant for all the paradigms of stimulation (train of 5—*P* = 0.008; train of 4—*P* = 0.01; train of 3—*P* = 0.009; and train of 2—*P* = 0.005) apart from train of 1 (*P* = 0.35).

Table 2 shows the median excitability difference and IR for the different trains and WHO grade tumors. Our Kruskall–Wallis analysis showed significant differences in the median RMT excitability difference between the groups (WHO grade 2, 3, and 4) in response to a train of 5 stimulation paradigm (*P* = 0.008), a train of 4 (*P* = 0.01), a train of 3 (*P* = 0.009), and a train of 2 paradigm (*P* = 0.005). No significant differences were found, however, for absolute RMTs (asleep or awake) and tumor grade. “Outliers,” or patients with abnormally high or low excitability differences were included in the analysis. We believed this was a better reflection of the biological reality in our data. Figure 3 is an illustration of the different groups by tumor grade and a box plot serves for visualization (via bar plots) of the median RMT excitability differences amongst the groups to the different stimulation trains. For trains of 5-2 pulses, an increasing median difference can be found.

No significant differences amongst the medians were found when sex, age, involvement of the M1-CST complex by tumor (location: frontal, parietal, insular, or temporal), oedema, or the presence of seizures (focal or generalized) were studied. Therefore, these factors we assumed were not confounders of our results.

**Fig. 1:**
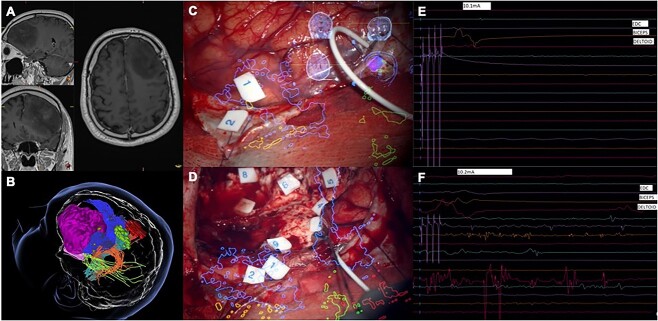
**WHO grade 2 asleep—awake excitability difference.** Example: (A) Structural imaging showing a diffuse glioma in the left frontal lobe (sagittal, coronal and axial T1-gadolinium enhanced images). (B) 3D preoperative modeling of preoperative mapping (magenta—tumor; dark blue—fronto-aslant tract; red—TMS-seeded CST for the upper limb; light blue—TMS-seeded corticospinal for the upper limb; orange—arcuate fasciculus; green—inferior fronto-occipital fasciculus; red dots—TMS positive responses for the lower limb; green dots—TMS positive responses for the upper limb. (C and D) Intraoperative imaging with overlay of tractography and TMS models (1 and 2—speech arrest; 3 and 4—dual task arrest; 5—initiation deficit; 6, 7, and 8—semantic errors; all cortical responses elicited at 4 mA and subcortical responses at 5 mA with low frequency stimulation). (E and F) Intraoperative motor threshold determinations during the asleep and the awake phase of the procedure showing an excitability difference of 0.1 mA.

**Fig. 2:**
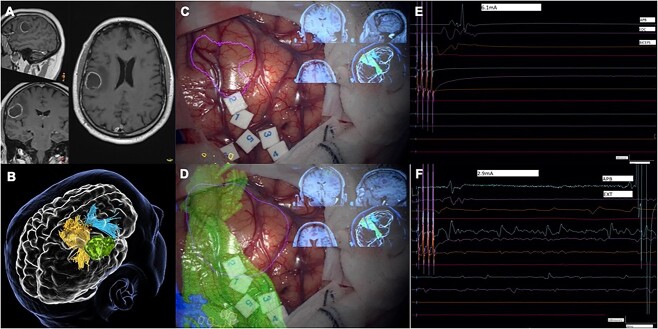
**WHO grade 4 asleep—Awake excitability difference.** Example: (A) Structural imaging showing a diffuse glioma in the left frontal lobe (sagittal, coronal and axial T1-gadolinium enhanced images). (B) 3D preoperative modeling of preoperative mapping (green—tumor; yellow—CST; blue—fronto-aslant tract; orange dots—TMS positive responses for the upper limb). (C and D) Intraoperative imaging with overlay of tractography and TMS models (CST in green in these images; 1 and 2—tongue (8 mA); 3 and 4—upper limb (8 mA); 5 and 6—upper limb (7 mA). (E and F) Intraoperative motor threshold determinations during the asleep and the awake phase of the procedure showing an excitability difference of 3.2 mA.

**Fig. 3:**
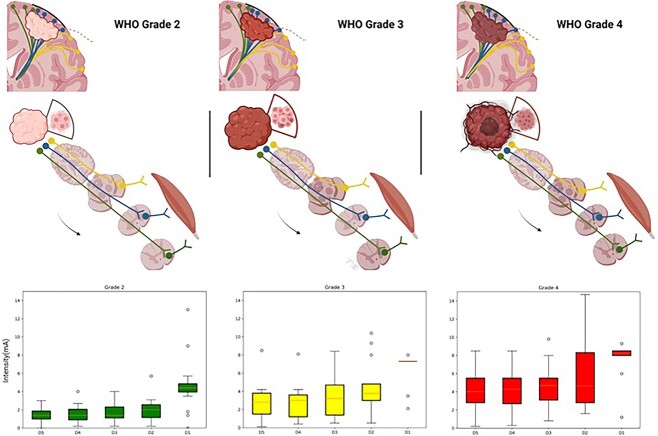
**Excitability differences between the asleep and awake states according to the paradigm of stimulation**. On the top, a schematic representation of motor eloquent gliomas affecting the M1-CST complex is demonstrated with different colors of the representing different functional specialization (green—lower limb; blue—upper limb; yellow—face). In the inferior half of the figure, different box plots graphs show the differences in the RMTs between the asleep and awake states according to the paradigm of stimulation per WHO grade. (*Illustration with Biorender).*

Our Spearman correlation analysis, to support the significant differences found, showed the following numerical trends: A significant positive correlation was found between RMT excitability difference and the tumor grade in our patients after cortical stimulation with a train of 5 pulses (R = 0.54, *P* = 0.00063), 4 pulses (R = 0.49, *P* = 0.002), 3 pulses (R = 0.51, *P* = 0.001), and 2 pulses (R = 0.54, *P* = 0.0007). Increasing differences between the RMTs in asleep and awake phases were observed progressing from grade 2 to grade 4 gliomas. Figures 1 and 2 document intraoperative examples of these differences in both low- and high-grade diffuse gliomas, respectively. We performed similar correlations between age and excitability difference in response to the different stimulation trains, which were all nonsignificant (*P* > 0.05 for all stimulation trains). Figure 3 shows boxplot graphs of median excitability difference and IR of RMTs in response to the different train pulse paradigms for each group of patients (WHO grade 2, 3, and 4).

**Fig. 4:**
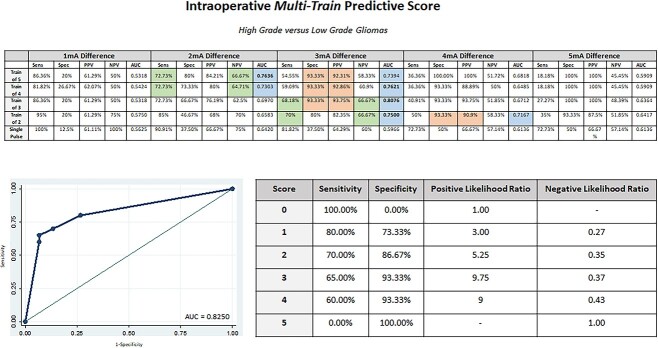
**Intraoperative multi-train predictive score**: Receiver operating characteristic analysis for different paradigms of stimulations considering progressive cortical RMT differences between asleep and awake phases between low- and high-grade glioma patients. Sensitivity, specificity, PPV, negative predictive value, and AUC are reported. Models with AUC > 0.70 were considered and an intraoperative multi-train predictive score was elaborated. Color code: green—Higher sensitivity and negative predictive value; orange—higher specificity and PPV; blue—models with AUC > 0.70; bold—best model for each stimulation paradigm.

Only differences of 2, 3, and 4 mA produced models with AUC > 0.70. For high-grade glioma prediction (versus low grade), 2 mA difference produced the model with higher AUC with train-of-5 stimulation paradigm and 3 mA difference for train-of-4, train-of-3, and train-of-2 stimulation paradigms (Fig. 4). Similar results were obtained for WHO grade 3 prediction (versus WHO grade 2) but no paradigm achieved an AUC > 0.70 in prediction of WHO grade 4 (versus WHO grade 3) (Table 3). As the paradigm stimulation delivers less energy to the brain, we observe a shift from 2-3 to 3-4 mA difference in the best models. When more than one model is considered for a single paradigm of stimulation, the lower difference is related with better sensitivity and negative predictive value (NPV) and the higher difference with a better specificity and PPV (Fig. 4 and Table 3). The combine score based on the best models for each paradigm of stimulation predict high grade glioma with AUC = 0.8250 (Fig. 4).

**Table 3:** **Excitability difference analysis between asleep and awake phases according to WHO grading.** Analysis for different paradigms of stimulations considering progressive cortical RMT differences between asleep and awake phases between WHO grade 2 and 3 (top) and WHO grade 3 and 4 (bottom) glioma patients. Sensitivity, specificity, PPV, negative predictive value, and AUC are reported. Models with AUC > 0.70 were considered and an intraoperative multi-train predictive score was elaborated. Color code: green—higher sensitivity and negative predictive value; orange—higher specificity and PPV; blue—models with AUC > 0.70; bold—best model for each stimulation paradigm.

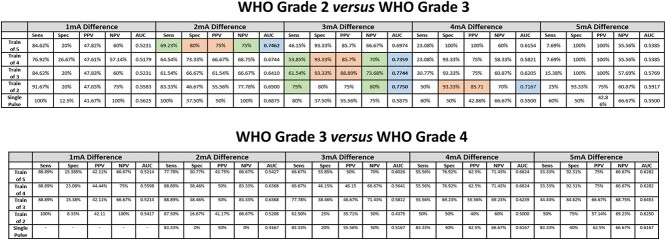

### Bayesian network analysis

Our Bayesian Network analysis showed that given an excitability difference above 3 mA between asleep and awake phases—strongest and more consistent threshold as per ROC analysis—the tumor had the highest probability of being high grade (75.8 versus 24.2% for low grade) with a probability of it being Grade 3 of 39.7% and Grade 4 of 36.14% (sensitivity of 54.5%, specificity of 93.3%, PPV of 92.3% and NPV of 58.3%, 5-pulse stimulation train).

If the excitability difference was below 3 mA, there is a higher probability of a tumor being WHO grade 2 than grade 3 or 4—48.8 versus 32 versus 19%—but the model cannot reliably predict low grade versus high grade glioma—48.8 versus 51.2% (sensitivity of 93.3%, specificity of 66.7%, PPV of 70% and NPV of 92.3%, 5-pulse stimulation train).

In summary, these results show that RMT excitability differences vary significantly between the groups (Kruskall Wallis), presenting a significant positive correlation between RMT excitability difference and WHO grade (Spearman correlation). A 3 mA cut-off in RMT excitability difference could be a marker to determine low versus high grade tumors (Bayesian analysis).

## Discussion

Our results suggest a progressive increase of motor cortical excitability with an increase in the WHO grading of diffuse infiltrative gliomas. This is supported by an increase in the RMT excitability difference of the motor cortex between the asleep and awake phases with different paradigms of RMT determination (5 to 2 pulses). Absolute RMT values, however, showed no significant differences between the groups. This shows RMT values are not different between different patients in both asleep and awake conditions but the individual difference per patient in RMT excitability between the asleep and awake phase is an independent marker of significant excitability change between awareness phases for that individual.

Grade 2 tumors showed similar RMTs in both stages to the different train stimulation paradigms, whereas grade 3 tumors showed a higher difference and grade 4 tumors, the highest. Figure 3 summarizes this concept and our main findings. These results are reproducible with different stimulation paradigms (5 to 2 pulses). We therefore assume that the results seen on cortical excitability and tumor grade were not influenced by the number of electrical pulses applied (no electrical pre-pulse facilitation effect) (Nitsche and Paulus 2000).

Our ROC analysis models suggest a difference between asleep and awake phases above 3 mA as the best predictor of tumor grading across different paradigms of stimulation—AUC = 0.8250 for the combined multi-train score. Our Bayesian Network analysis confirms these findings as an excitability difference above 3 mA confers a probability of 75.8% of a glioma being a high grade. When a difference below 3 mA is considered, the results support the progressive impact of tumor biology in the cortical excitability as there is a progressive decrease of probability from WHO grade 2 to WHO grade 4–48.8 versus 32 versus 19%—as it was suggested by previous work using TMS (Lavrador et al. 2020). Nevertheless, TMS-derived data from previous published work (Lavrador et al. 2020; Neville et al. 2021) cannot be directly compared with this intraoperative neuromonitoring derived data as their methodology is significantly different. The TMS literature compare interhemispheric differences in a single physiological state (awake) whilst this results use single-hemisphere data (tumor side) in different physiological states (asleep versus awake) in the same patient.

Tumor-neuroglial synapses are at the center of the driving theories underlying cortical excitability modulation with tumor grading (Sontheimer 2008; Pei et al. 2020; Wirsching and Weller 2020; Venkatesh et al. 2019). Venkataramani et al. (2019) reported that glioma cells promote neuro-glional synapses which are mediated by functional chemical links between presynaptic neurons and postsynaptic glioma cells therefore raising awareness about the impact of infiltrative tumor cells within the peri-motor areas.

Motor cortex excitability can be modulated with awareness. The changes we recorded in the RMT during the awake phase of surgery could be accounted for by voluntary muscle tone activation. Therefore, the enhanced excitability could reflect increased voluntary movements during the awake phase, which could be very variable from patient to patient. Our methodology attempted to decrease the chance of this. Awake RMTs were done at the very early stages of awareness, when only a slight increase in baseline EMG was seen alongside an increase in the fast frequency power of the electroencephalography(EEG), with no voluntary movements were recorded. Therefore, all our patients (irrespective of tumor grade) presented similar motor conditions to record awake RMTs. Sequential train stimulation can hypothetically induce long-term-potentiation (LTP) (Landau et al. 1964; Purpura and McMurtry 1965). Our stimulation paradigm, despite the longer trains, was never as prolonged as 10–30 min to induce LTP. Therefore, we believe that modulation of the motor cortex would not be sustained in time. This was an important consideration when determining thresholds during the awake phase of the procedure.

This study has some limitations. Our sample size is small as this is a proof-of-concept study. This may affect future applications of the technique and larger prospective studies should be carried out to establish our findings. The WHO grading imbalance in the patients included in this study reflects the eligibility for awake surgery. This paradigm is changing (Wirsching and Weller 2020) and we will probably see an inverted correlation in future publications in this field. Also, we used clinical assessment for determination of awake state and not only EEG-based criteria. This is supported by the literature (Zetterlund et al. 2016) to account for the wide interindividual variability of these techniques (Kaskinoro et al. 2011). We did not study the effects of tissue impedance in our sample, which could be something future studies on the matter could focus on. This study also did not study subcortical thresholds during asleep and awake phases of surgery. Whether the results of our study modify subcortical excitability is something that was not our focus.

Despite the above, this is the first study that uses the information provided by intraoperative neurophysiology to aim at predicting tumor grading based on motor excitability. The actual diagnosis was performed after surgery and the investigators were blinded to WHO grade determination when calculating the excitability RMTs and differences. In the future and after external validation, intraoperative-derived tumor grading information can empower surgical teams to adapt their surgical strategies and meet the patients’ needs.

## Conclusion

Our study suggests that the higher the difference in the RMTs between asleep and awake states, the higher the WHO tumor grading in gliomas regardless the patients’ demographics prior to surgical intervention. A threshold of 3 mA is related with the best predictive models across different patterns of M1-CST stimulation.

## Abbreviations

MEP: motor evoked potential

WHO: World Health Organization

CST: corticospinal tract

DNA: deoxyribonucleic acid

RMT: resting motor threshold
